# Mitochondrial localization of SESN2

**DOI:** 10.1371/journal.pone.0226862

**Published:** 2020-04-14

**Authors:** Irina E. Kovaleva, Artem V. Tokarchuk, Andrei O. Zheltukhin, Alexandra A. Dalina, Grigoriy G. Safronov, Alexandra G. Evstafieva, Konstantin G. Lyamzaev, Peter M. Chumakov, Andrei V. Budanov

**Affiliations:** 1 Belozersky Institute of Physico-Chemical Biology, Moscow, Russia; 2 School of Biochemistry and Immunology, Trinity Biomedical Sciences Institute, Trinity College Dublin, Dublin, Ireland; 3 Center for Precision Genome Editing and Genetic Technologies for Biomedicine, Engelhardt Institute of Molecular Biology, Russian Academy of Sciences, Moscow, Russia; 4 Faculty of Bioengineering and Bioinformatics, Lomonosov Moscow State University, Moscow, Russia; Roswell Park Cancer Institute, UNITED STATES

## Abstract

SESN2 is a member of the evolutionarily conserved sestrin protein family found in most of the *Metazoa* species. The SESN2 gene is transcriptionally activated by many stress factors, including metabolic derangements, reactive oxygen species (ROS), and DNA-damage. As a result, SESN2 controls ROS accumulation, metabolism, and cell viability. The best-known function of SESN2 is the inhibition of the mechanistic target of rapamycin complex 1 kinase (mTORC1) that plays a central role in support of cell growth and suppression of autophagy. SESN2 inhibits mTORC1 activity through interaction with the GATOR2 protein complex preventing an inhibitory effect of GATOR2 on the GATOR1 protein complex. GATOR1 stimulates GTPase activity of the RagA/B small GTPase, the component of RagA/B:RagC/D complex, preventing mTORC1 translocation to the lysosomes and its activation by the small GTPase Rheb. Despite the well-established role of SESN2 in mTORC1 inhibition, other SESN2 activities are not well-characterized. We recently showed that SESN2 could control mitochondrial function and cell death via mTORC1-independent mechanisms, and these activities might be explained by direct effects of SESN2 on mitochondria. In this work, we examined mitochondrial localization of SESN2 and demonstrated that SESN2 is located on mitochondria and can be directly involved in the regulation of mitochondrial functions.

## Introduction

Sestrins belong to the evolutionarily-conserved protein family found in most of the species of the animal kingdom [1]. While invertebrate genomes contain only one gene encoding sestrin, genomes of vertebrates contain three sestrin genes (SESN1-3). Sestrins are stress-responsive proteins that play a significant role in the regulation of cell viability through the control of reactive oxygen species (ROS) and the regulation of metabolism [1]. Although sestrins are dispensable in embryogenesis, they support homeostasis by suppressing the accumulation of age-related damages in different tissues of an organism. Notably, our studies demonstrated that inactivation of sestrin in *Drosophila* leads to deterioration of muscle tissue and excessive accumulation of lipids and carbohydrates [2, 3]. The inactivation of sestrin (cSesn) in *C*. *elegans* shortens the lifespan of the animals and weakens their resistance to stresses [4, 5]. Moreover, inactivation of sestrin family members in mammals facilitates the development of metabolic syndrome, cardiac malfunction, some types of cancer, and muscle atrophy [3, 6–10].

SESN2 is the best-characterized member of the sestrin family. The expression of the *SESN2* gene is activated by several transcription factors including the tumor suppressor protein p53, the regulator of antioxidant response NRF2, and the regulator of integrated stress response ATF4 [1, 11–13] supporting the potential role of SESN2 in the regulation of cellular homeostasis under these stress conditions [14]. Our previous works demonstrated that SESN2 modulates cell viability in response to stress, and the outcome of its activation depends on the type of stress [11, 12, 15, 16]. According to our data, SESN2 protects from ischemia and oxidative stress but can support cell death in response to certain types of DNA-damage and pro-apoptotic cytokines [11–13, 17]. One of the major functions of sestrins is the suppression of the mechanistic target of rapamycin complex 1 (mTORC1) kinase [18, 19]. Sestrins inhibit mTORC1 through direct interaction with the GATOR2 protein complex, composed of proteins Mios, WDR24, WDR59, Seh1L, and Sec13 [20–22]. GATOR2 inhibits the GATOR1 complex, containing DEPDC5, NPRL2, and NPRL3 proteins. GATOR1 works as a GTPase activating protein for the small GTPases RagA and RagB [23], the components of RagA/B:RagC/D heteromeric complexes that in the active form interact with mTORC1 and translocate the latter to the lysosomal surface where mTORC1 is activated by the small GTPase Rheb [24]. Recent studies showed that the interaction between SESN1/2 and GATOR2 complex could be negatively regulated by amino acid leucine that binds the leucine-binding domain of sestrins and disrupts the interaction between SESN1/2 and GATOR2, facilitating inhibition of GATOR1 by GATOR2, which leads to mTORC1 activation [25]. However, many types of stress may stimulate the formation of SESN2-GATOR2 complexes through the increased expression of sestrins and, possibly, via some posttranslational modifications [20, 26]. Although GATOR1 plays a major role in the suppression of mTORC1, this complex is also involved in the regulation of mitochondrial homeostasis and cell death in response to DNA damage [27]. Autophagy plays a significant role in the regulation of cell viability after stresses. SESN2 promotes mitophagy, a specific form of autophagy, either through the inhibition of mTORC1 or through some other mechanisms, such as interaction with the autophagy receptor SQSTM1/p62 and the E3-ubiquitin ligase Rbx1 [18, 28].

SESN2 may also be involved in the regulation of metabolism and cell death through the control of mitochondrial functions. We have previously demonstrated that SESN2-deficient mouse embryonic fibroblasts have diminished ATP production comparatively to wild type counterparts [13, 29]. Mitochondria is the primary source of ATP in cells, and SESN2-deficient MEFs have a lower respiration rate than the control cells [13], suggesting a potential role of sestrins in the mitochondrial regulation. However, whether sestrins regulate mitochondrial processes through their direct association with mitochondria or these effects are mediated by some additional factors are yet to be identified. The focus of these studies was to determine whether SESN2 might play a direct role in the regulation of mitochondrial functions such as mitochondrial respiration and cell death through interaction with mitochondria. In this work, we performed an analysis of intracellular localization of SESN2 in normal and stress conditions and demonstrated that SESN2 could be co-localized with mitochondria, potentially regulating the activity of this organelle.

## Materials and methods

### Cell culture conditions

Human lung adenocarcinoma A549 cells, human osteosarcoma U2OS cells, and SV40 T antigen-immortalized human lung fibroblasts (MRC5/T) were maintained in High Glucose DMEM containing 25 mM glucose and 10% FBS (Hyclone) or in the galactose medium composed of glucose-free DMEM supplemented with 10 mM galactose, 2mM glutamine (4 mM final) and 10% dialyzed FBS (Hyclone) until they reached 70–80% confluence. To induce glucose starvation, cells were rinsed with glucose-free DMEM and further incubated in glucose-free DMEM supplemented with 10% dialyzed FBS for 12 h. The cells were kept in 5% CO_2_ at 37°C.

### Preparation of mitochondrial and cytosolic fractions

A549 or MRC5/T were harvested from 150 mm dishes and homogenized in 550 μl of Mitochondrial Isolation Buffer (MIB) (220 mM mannitol, 70 mM sucrose, 10 mM Hepes pH 7.5, 1 mM EDTA and 1% CompleteTM protease inhibitor cocktail (Roche)) by passing the cell mixture through a 26-gauge needle at 4°C (50–100 strokes). The homogenate was centrifuged at 1000 g for 10 min at 4°C to remove the nuclei and the intact cells. The centrifugation step was repeated, and the supernatant was centrifuged at 9000 g for 10 min at 4°C. The pellet was washed twice with 1ml of MIB. Approx. 5 μg of protein of the post mitochondrial supernatant and the pellet (crude mitochondria) were loaded per lane of 10% SDS/PAGE gel for Western blot analysis.

### Isolation of mitochondria with anti-TOMM22 magnetic microbeads

Mitochondria were isolated using Mitochondrial Isolation Kit human (Miltenyi Biotec) according to the instructions of the manufacturer. In brief, A549 cells were harvested at approx. 90% confluency and washed twice with PBS. Then cells (1x10^7^) were lysed in 1 ml of ice-cold lysis buffer with Complete Protease Inhibitor Cocktail (Roche) by shearing through a 26 G needle for 30–40 times. After cell disruption, 9 ml of binding buffer and 50 μl of anti-TOMM22 microbeads for magnetic labeling of the mitochondria were added to the crude cell lysates. The labeling proceeded for 1 hour at 4°C with gentle shaking by the Bio RS-24 mini rotator. Subsequently, the suspension was loaded onto a preequilibrated MACS column (Miltenyl Biotec) placed in a MACS Separator (Miltenyl Biotec). After washing the column with binding buffer, it was removed from the MACS Separator and the retained mitochondria were eluted with 1.5 ml of the binding buffer and sedimented at 13000 g for 1 min. Then the mitochondrial pellet was washed twice with the storage buffer (0.32 M sucrose, 1 mM EDTA, and 10 mM Tris–HCl) and finally resuspended in the 50 μl of the same buffer or MIB.

### Immunoblot analysis

5 μg of protein per lane was separated on 10% SDS/PAGE gel and transferred onto a Hybond ECL® (enhanced chemiluminescence) membrane (Amersham Biosciences). After blocking with 5% milk for 1 h, the membrane was incubated overnight at 4°C with primary antibodies diluted 1:1000 followed by a further 1 h incubation with the corresponding horseradish peroxidase-conjugated secondary antibody (Bio-Rad) at a 1:10000 dilution, and the signal was detected with the ECL® Western blotting detection system (Amersham Biosciences). The following primary antibodies were used for immunoblotting: Anti-AKAP1 (D9C5), Anti-COX4 (3E11), Anti-Mios (D12C6), Anti-Sestrin2 (D186) were from Cell Signaling Technology; Anti-Actin (ab3280) was from Abcam; Anti-GAPDH (G9545) was from Sigma-Aldrich. Secondary antibodies were from Santa Cruz Biotechnology: m-IgGκ BP-HRP (sc-516102) and mouse anti-rabbit IgG-HRP (sc-2357).

### Immunoprecipitation

Mitochondria isolated from three confluent 150 mm dishes were resuspended in the 200 μl of lysis NP40 buffer (0.3% NP40, 150mMNaCl, 50mMTris pH 7.5) supplemented with protease and phosphatase inhibitors (cOmplete Roche), incubated for 20 min on ice and centrifuged in a refrigerated centrifuge at 10000 RPM for 15 min. 10 μl Anti-SESN2 antibodies (41-K, Santa Cruz Biotechnology) or anti-AKAP1 antibodies (D9C5, Cell Signaling Technology) and 10 μl of protein A/G agarose (Pierce) 50% slurry in the lysis buffer were added to the supernatant, and then the mixture was incubated for 4 h on a rotating platform. The beads were washed 4 times with 1 ml NP40 buffer and reconstituted in the 50 μl of 2X electrophoresis loading buffer, and then the probes were heated at 100°С for 5 min, centrifuged 1 min at maximum speed, and loaded on a gel for subsequent immunoblot analysis.

### Proteinase K treatment

Isolated mitochondria were resuspended in MIB and treated with Proteinase K (50 μg/ml) for 20 min on ice. The reaction was stopped by incubation with PMSF (1mM) for 10 min on ice, and the probes were analyzed by immunoblotting.

### Fluorescent microscopy

To determine mitochondrial co-localization of SESN2, U2OS cells stably expressing GFP- tagged SESN2 (SESN2-GFP) were exposed to 10 μM FCCP treatment for 24h and, before the fluorescent microscopy procedure, were incubated with 100 nM MitoTracker Red (CMXRos, Invitrogen) in DMEM for 30 min at 37°C to stain mitochondria. Cells were analyzed using a Nikon confocal microscope C2 on inverted Eclipse Ti (Nikon) equipped with 60X/1.4 objective.

### Analysis of respiration in *C*. *elegans*

#### Nematode strains

The wild-type WT (N2) and the cSesn^-/-^ (RB2325, genotype ok3157) animals with a 535 bp deletion in exon 3 of *cSesn* gene were purchased from the Caenorhabditis Genetics Center (CGC) of University of Minnesota and maintained on the sterile NGM (Nematode Growth Medium) containing 2% agarose, 0.3% NaCl, 1 mM MgSO_4_, 1 mM CaCl_2_, 0.25 M phosphate buffer, 0.3% bactopeptone, and 0.05% cholesterol as described [5]. A night culture of *E*. *coli* (strain OP50, Uracil auxotroph) was spread out on agar beds of Petri dishes and the bacteria were grown overnight at 37°C. All experiments with nematodes were carried out at 20°C.

#### Synchronization of nematodes

To prepare a synchronous culture of nematodes, adult animals were treated with a solution containing 1% NaClO and 0.5 M KOH for 10 minutes. The released nematode eggs were washed twice in M9 medium [5] and incubated overnight at a temperature of 20°C in M9 medium. The next day, nematodes at the L1 stage were placed onto Petri dishes with seeded OP50.

#### Sample preparation

Synchronized L4 animals were washed from OP50 plates with M9 medium, rinsed twice, and rested in M9 medium for 20 minutes to remove bacteria from their guts to avoid the potential effects of bacteria on oxygen consumption rate (OCR) in *C*. *elegans*. Then nematodes were resuspended in unbuffered EPA water (60 mg MgSO_4_x7H_2_O, 60mg CaSO_4_x2H_2_O, 4 mg KCl, ddH_2_O to 1L) one worm per microliter of the solution (confirmed by counting the number of worms in 20 μl drops). 75 microliters of the solution containing worms were distributed into each well of a 24-well Seahorse utility plate using tips rinsed in 0.1% Triton X-100 to prevent worms sticking. The final volume of each well was then brought to 525 μl with unbuffered EPA water. Four wells in an assay were left as blanks. 75 μL unbuffered EPA water with 80 μM FCCP (3.2% DMSO) and 320 mM sodium azide were then loaded into the injection ports of the Seahorse cartridge. During Seahorse assay, after injection, each drug solution was diluted eight times, and final concentrations of drugs were 10 μM FCCP and 40 mM sodium azide.

### Analysis of mitochondrial respiration

The analysis of mitochondrial respiration in worms was done as described previously [30]. Seahorse software WAVE was set up for 16 cycles, consisted of a mix cycle for oxygenation of the wells (1 min), a waiting period to make worms settled down (3 min), and a measurement of oxygen levels (3 min). Basal OCR was measured in eight cycles, then FCCP or azide was injected, followed by an examination of OCR in eight more cycles. All OCR values were normalized per worm number as counted under the microscope. Worms treated with the mitochondrial uncoupler FCCP and complete respiratory inhibitor sodium azide were used for the determination of maximal respiratory capacity, spare respiratory capacity, and non-OXPHOS OCR. The experiment was done twice with 5 technical repeats for each experiment with a similar outcome.

### Assessment of mitochondrial respiration of A549 cells

The mitochondrial respiration of A549 cells was measured using the Agilent Seahorse XF24 MitoStress test. One day before the experiment, the cartridge was hydrated according to the manufacturer’s instructions, and cells were seeded at a density of 28000 cells per well. Before the experiment, cells were washed with XF base medium supplied with 25 mM glucose, 2 mM glutamine, and 2 mM pyruvate and incubated in 0.5 ml of the same medium for 30 min at 37^o^ without CO_2_. The injections were as following: 1.5 μM oligomycin, 400 μM 2,4-dinitrophenol (2,4-DNP), the mix of 1 μM rotenone and 1 μM antimycin A. The protocol had been changed: 1 min mixing, 2 min waiting, 3 min measuring. OCR in each well was normalized by the total protein amount measured using the Bradford assay. The experiment was performed with 5 replicates and was repeated for three times. OCR values (pmol/min) were normalized by total protein amount (μg).

### Statistical analysis

Data were processed using STATISTICA and software package (http://www.statistica.io). The comparisons of the two mean values were estimated using two-tailed Student’s t-test, *p≤0.05, **p≤0.01, ***p≤0.001.

## Results

### Purification of SESN2 in the mitochondrial fraction

**Fig 1:**
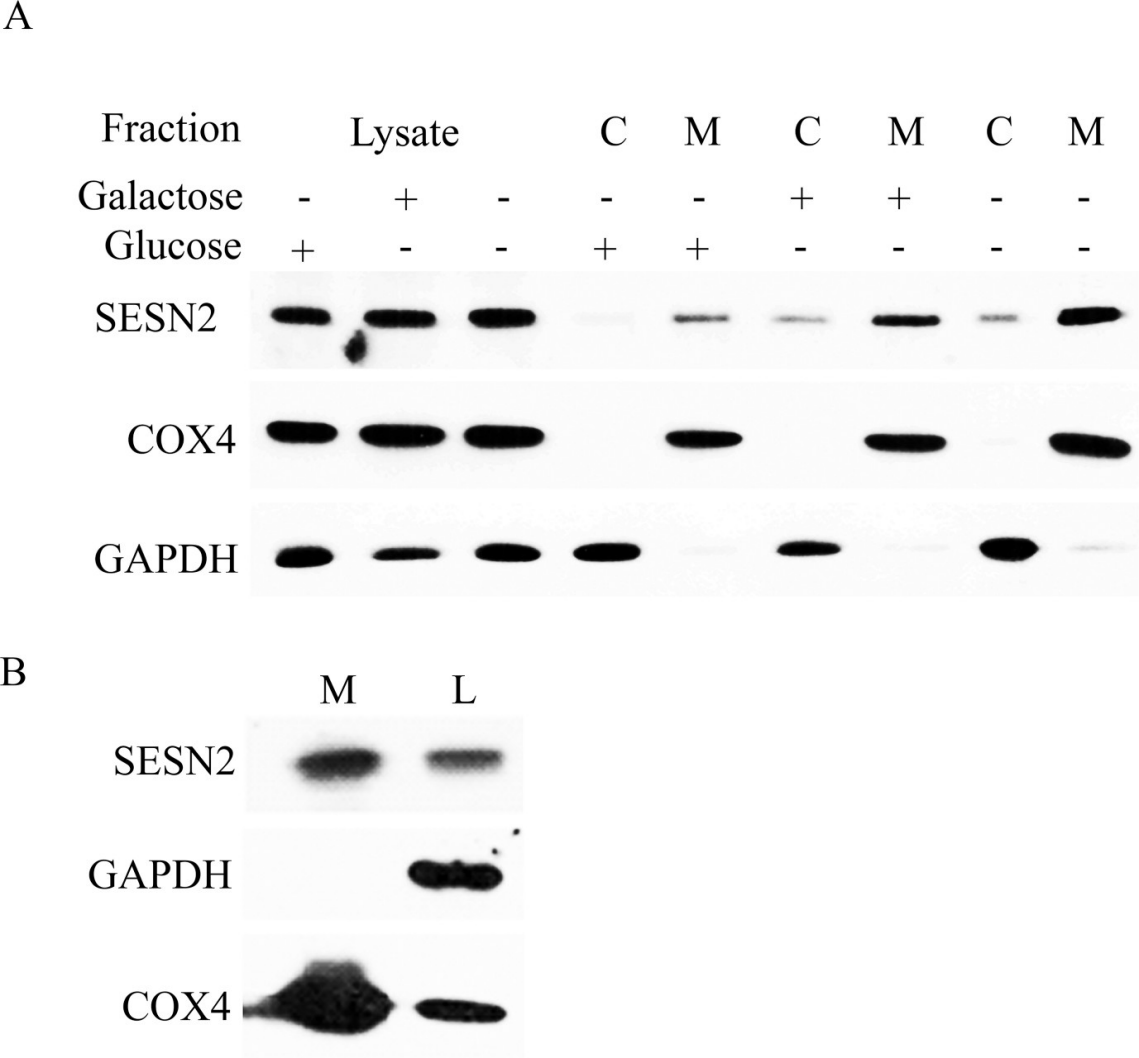
SESN2 is co-purified with mitochondria. (A) A substantial amount of endogenous SESN2 is detected in the fraction of crude mitochondria. A549 cells grown in the indicated conditions were fractionated by differential centrifugation to get crude mitochondria and cytosolic fractions. Equal protein amounts (5 μkg) of the whole cell lysate, the cytosolic fraction (C), and the mitochondria fraction (M) were analyzed by immunoblotting with anti-SESN2 antibody to detect endogenous SESN2. The mitochondria inner membrane protein COX4 and cytosolic protein GAPDH were used as positive and negative controls of mitochondrial and cytoplasmic samples to define mitochondria-localized proteins. A549 cells were incubated in the glucose-rich medium, in the medium, where glucose was replaced with galactose, or in the glucose-free medium. (B) SESN2 is detected with anti-SESN2 antibody in mitochondria purified with magnetic beads bearing an antibody against the mitochondrial outer membrane protein TOMM22. Mitochondria were isolated from A549 cells grown in galactose-containing media. M–purified mitochondria, L–whole cell lysate. For each sample, 5 μg of protein from each fraction was loaded and analyzed by immunoblotting with anti-SESN2, anti-GAPDH, and anti-COX4 antibodies.

To analyze the localization of SESN2, we performed differential centrifugation of cellular lysates to get mitochondrial (heavy membrane fraction) and cytosolic (light membrane fraction) as previously described [31]. As stress conditions may affect the localization of the protein, we analyzed lysates from the cells kept in a control condition, incubated in glucose-free medium, or incubated in the medium where glucose was replaced with galactose. Both stress conditions lead to a deficiency in energy production. However, glucose starvation can also cause ER stress and oxidative stress [1]. Western blot analysis of cytosol and membrane fractions demonstrated that a considerable portion of the SESN2 protein was co-purified with the heavy membrane fraction enriched with mitochondria (Fig 1A). We also observed that the amount of SESN2 protein co-purified with the membrane fraction was increased in stress conditions that may indicate that SESN2 is accumulated on the mitochondria to regulate metabolic activities of these organelles or cell death. The analysis of samples indicates that the quality of our preparation was quite high as we were not able to observe the mitochondrial protein COX4 (cytochrome C oxidase subunit 4) in the cytosolic fraction and the cytosolic protein GAPDH in the mitochondrial fraction (Fig 1A). As we used one-tenth of the mitochondrial fraction and one-hundredth of the cytosolic fraction for the loading on a gel, we can conclude that approximately equal amount of protein can be found in the cytoplasm and on the mitochondria according to the immunoblotting data (Fig 1A).

Although differential centrifugation allows us to obtain enriched mitochondrial fraction, it can also contain some contaminants from other membrane organelles. To perform more accurate purification of the mitochondrial fraction to confirm mitochondrial localization of SESN2, we performed high-affinity purification of mitochondria using magnetic beads bearing antibodies against mitochondrial outer membrane protein TOMM22. The effectiveness of this approach was verified in several recent studies and is considered to be of full value as an alternative to the ultracentrifuge method [32–34]. We observed that a considerable amount of SESN2 was co-purified with the mitochondrial fraction supporting the previous observation that the protein is associated with a mitochondrial fraction (Fig 1B). The high quality of purification of the mitochondrial fraction is supported by the fact that we observe strong enrichment of the mitochondrial protein COX4, but do not detect cytosolic GAPDH protein in the mitochondrial fraction (Fig 1B).

### SESN2-GFP fused protein is co-localized with a mitochondrial marker

**Fig 2:**
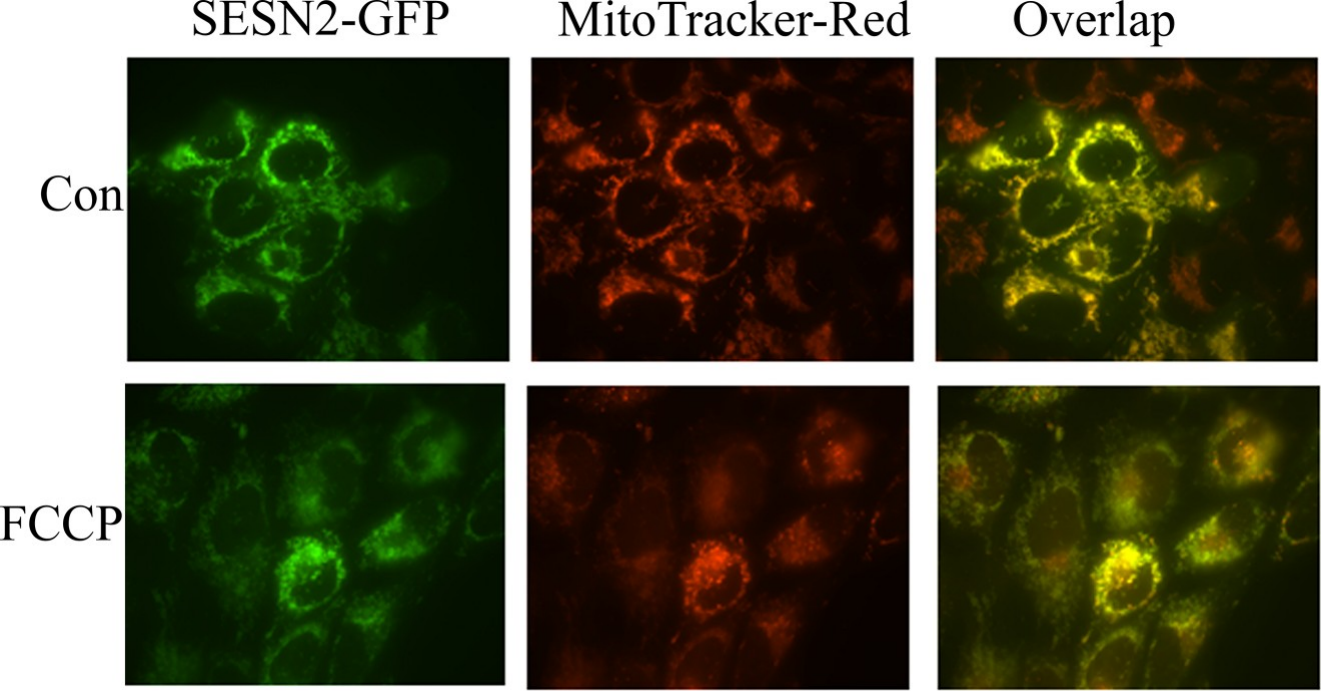
SESN2-GFP colocalizes with mitochondria. Confocal analysis of U2OS cells stably expressing SESN2-GFP fusion protein under basal conditions and upon treatment with 10 μM FCCP for 24 h. Mitochondria were stained with MitoTracker Red.

To confirm that SESN2 can be located on mitochondria, we generated a lentiviral expression construct where SESN2 is fused with fluorescent GFP protein, as previously described [12]. U2OS cells were infected with the SESN2-GFP expressing recombinant lentivirus and treated with mitochondrial uncoupler FCCP to disrupt mitochondrial potential and inhibit mitochondrial respiration. To analyze the localization of the SESN2-GFP protein under normal conditions or after FCCP treatment, the cells were incubated in the presence of Mitotracker Red, and protein localization was assessed by fluorescent microscopy. A substantial portion of SESN2-GFP was co-localized with the mitochondrial marker regardless of FCCP treatment, indicating that a considerable amount of SESN2-GFP protein is located on mitochondria, and this effect does not depend on the mitochondrial respiration (Fig 2).

### SESN2 is co-localized with GATOR2 complex on mitochondria

**Fig 3:**
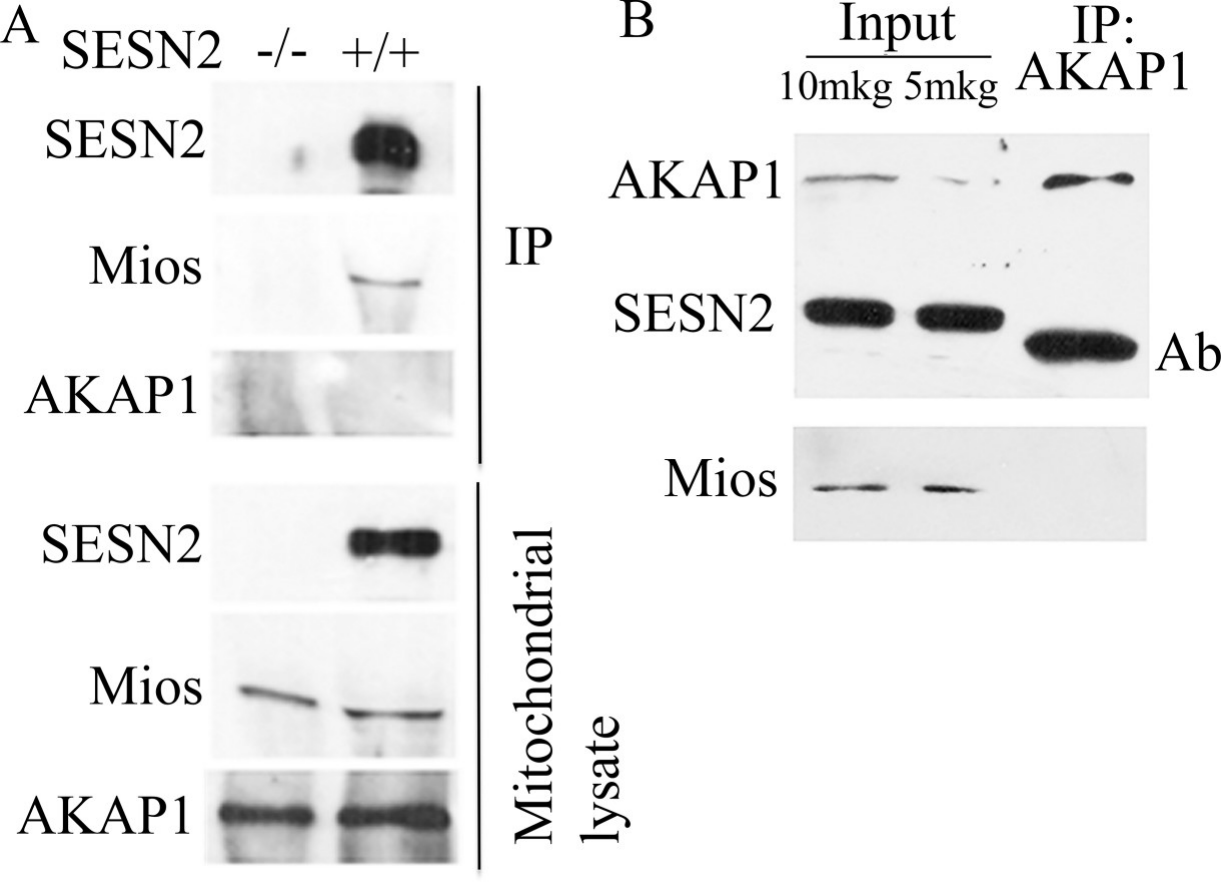
Mios coimmunoprecipitates with SESN2 from mitochondrial lysates. (A) immunoprecipitation of SESN2 from mitochondrial lysates. Mitochondria were isolated from control A549 cells or A549 *SESN2*^*-/-*^ cells after incubation in galactose media. SESN2-immunoprecipitates and mitochondrial lysates were analyzed by Western blot with anti-SESN2, anti-Mios, and anti-AKAP1 antibodies. (B) Immunoprecipitation of AKAP1 from mitochondrial lysates. Mitochondria were isolated from U2OS cells. Mitochondrial lysate and AKAP1-immunoprecipitate were analyzed with anti-SESN2 and anti-AKAP1 antibodies.

We and others have previously shown that GATOR2 is the critical partner of sestrins. However, the precise localization of this protein complex in the cell is not well-established [20, 21]. We analyzed whether Mios, a component of the GATOR2 complex, can be detected in the mitochondrial fraction. As observed in our studies, mitochondrial fraction contains Mios as well as SESN2 proteins (Fig 3A). We did not see any difference in the amount of Mios in the mitochondrial fraction from control and SESN2-deficient cells, indicating that mitochondrial localization of Mios does not require SESN2 (Fig 3A).

To determine whether SESN2 interacts with Mios on the mitochondria, we immunoprecipitated endogenous SESN2 from mitochondrial fraction using an anti-SESN2 antibody as previously described [20] and analyzed the presence of Mios in the SESN2 immunoprecipitates. As a negative control, we also analyzed mitochondrial lysates from the cells where SESN2 was knocked out with CRISPR/Cas9 [35]. While we were able to detect Mios in the immunoprecipitates from control cells, indicating that these proteins interact on mitochondria, we did not observe any presence of Mios as well as SESN2 protein in the immunoprecipitated samples from SESN2-deficient cells (Fig 3A).

It was previously reported that SESN2 is capable of interacting with mitochondrial AKAP1 protein on mitochondria and controlling mTORC1 via the AKAP1-dependent mechanism [36]. We analyzed whether AKAP1 is present in the mitochondrial fraction and in the SESN2 immunoprecipitates. While we observed a notable amount of AKAP1 protein in the mitochondrial fraction, we did not detect it in the SESN2 immunoprecipitates, indicating that it is unlikely that endogenous SESN2 interacts with AKAP1 (Fig 3A). We also performed an analogous experiment using anti-AKAP1 antibodies but were not able to detect either SESN2 or Mios in the AKAP1 immunoprecipitate (Fig 3B).

### GATOR2 affects mitochondrial localization of SESN2

**Fig 4:**
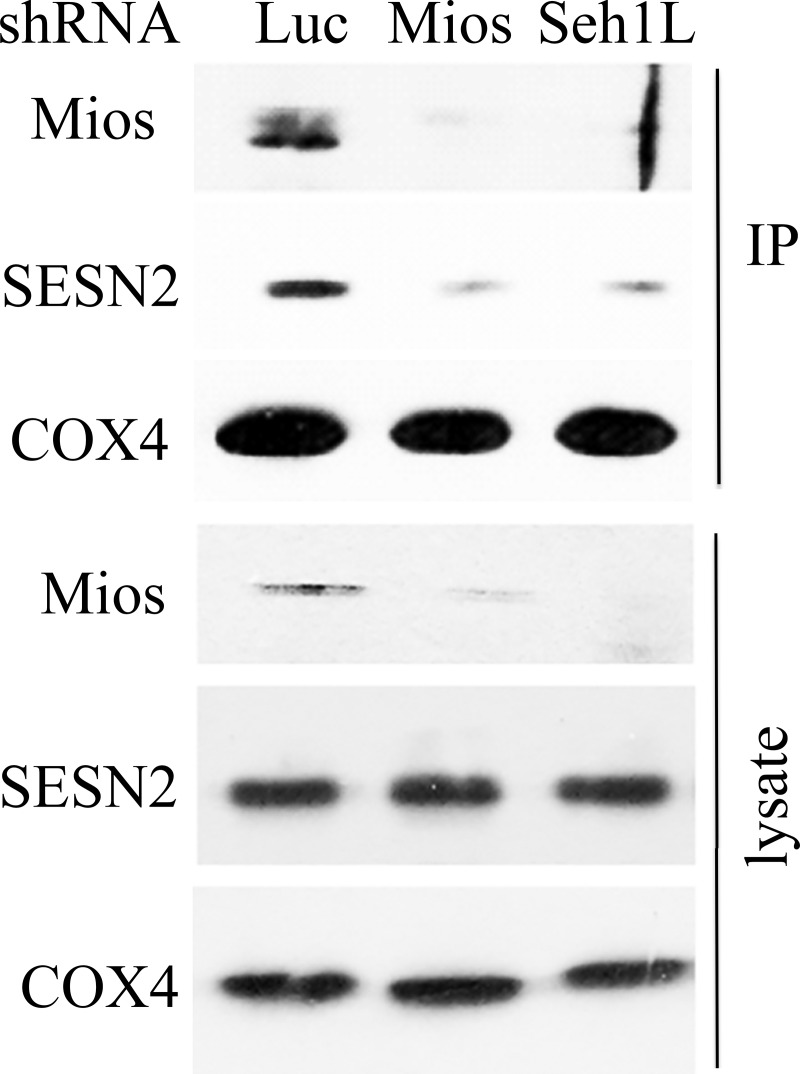
Downregulation of GATOR2 components Mios and Seh1L influences the accumulation of SESN2 in the mitochondrial fraction. Mitochondria were isolated with magnetic beads conjugated to anti-TOMM22 antibodies from A549 cells, expressing shLuc, shMios, and shSeh1L constructs, that were incubated in galactose-containing media.

We also determined whether mitochondrial localization of SESN2 depends on the GATOR2 complex. We generated cells where expression of either Mios or SehlL was silenced by shRNA lentivirus [20]. Both shRNAs strongly downregulate the expression of Mios (Fig 4) as it was previously reported [37]. In the cells where expression of GATOR2 proteins was silenced by shRNA we observed that amounts of SESN2 in mitochondrial fraction were notably decreased, indicating that GATOR2 proteins support mitochondrial localization of SESN2 (Fig 4).

### SESN2 is localized on the outer membrane of mitochondria or in the intermembrane space

**Fig 5:**
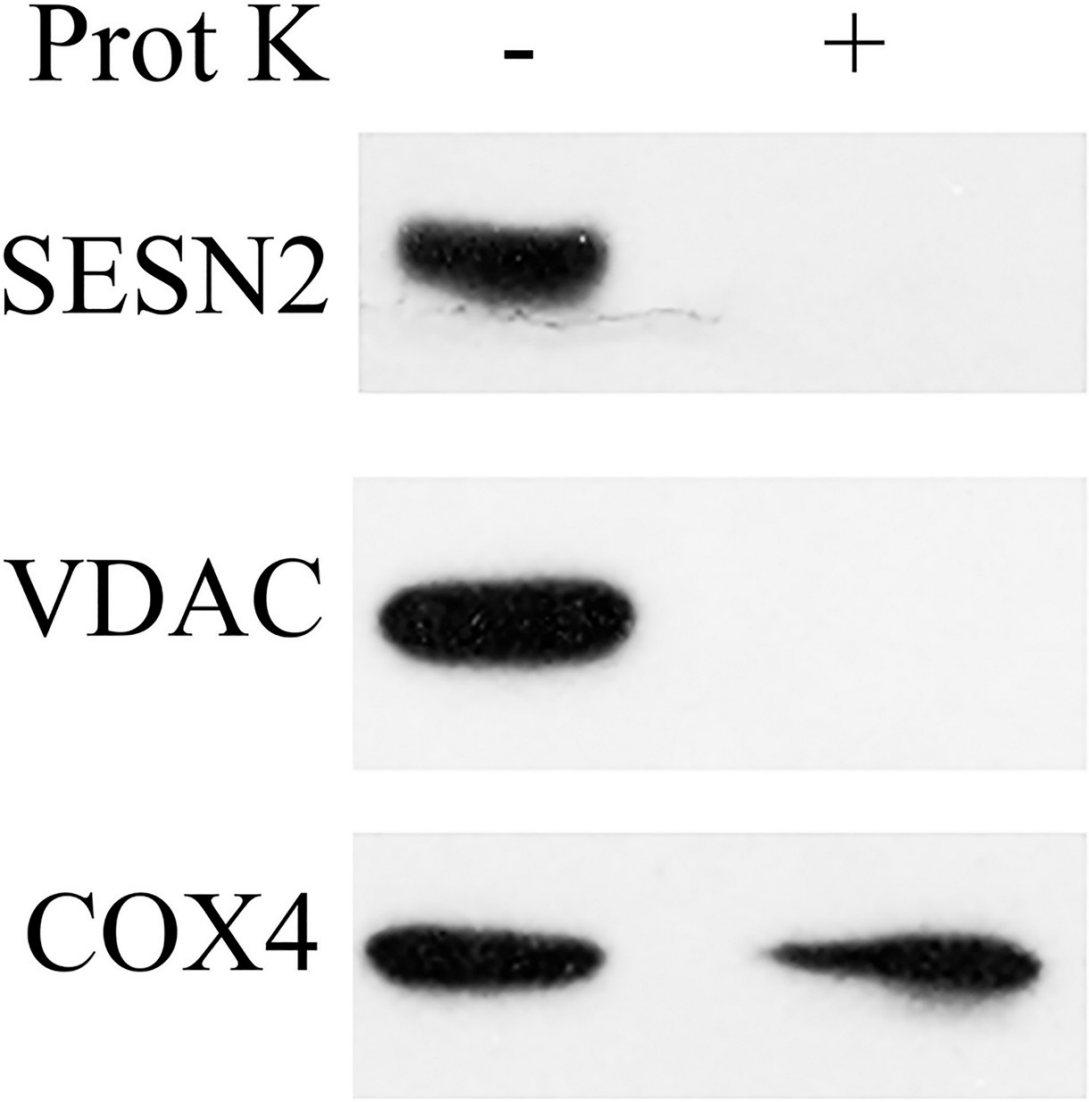
SESN2 is associated with the outer mitochondrial membrane. The mitochondrial fraction obtained from A549 cells was treated with 50 mg/ml proteinase K for 20 min on ice, and samples were subjected to immunoblot analysis to detect endogenous SESN2. VDAC1, and COX4 were used as markers for mitochondrial outer membrane and inner membrane, respectively.

To analyze whether SESN2 is transported inside of mitochondria or is located on the mitochondrial surface or in the intermembrane space we treated isolated mitochondria with proteinase K. We analyzed the presence of SESN2 in the untreated and proteinase K-treated mitochondria and observed that treatment with proteinase K led to disappearance of SESN2 from the mitochondrial fraction. A similar effect was observed for the voltage-dependent anion channel 1 (VDAC1) protein located on the outer mitochondrial membrane [38]. At the same time, we did not observe any changes in the amount of COX4, the component of the respiratory chain, embedded in the inner mitochondrial membrane [39]. Therefore, we concluded that SESN2 is located on the surface of mitochondria or in the intramembrane space (Fig 5).

### Sestrins regulate mitochondrial respiration in C. elegans and A549 cells

**Fig 6:**
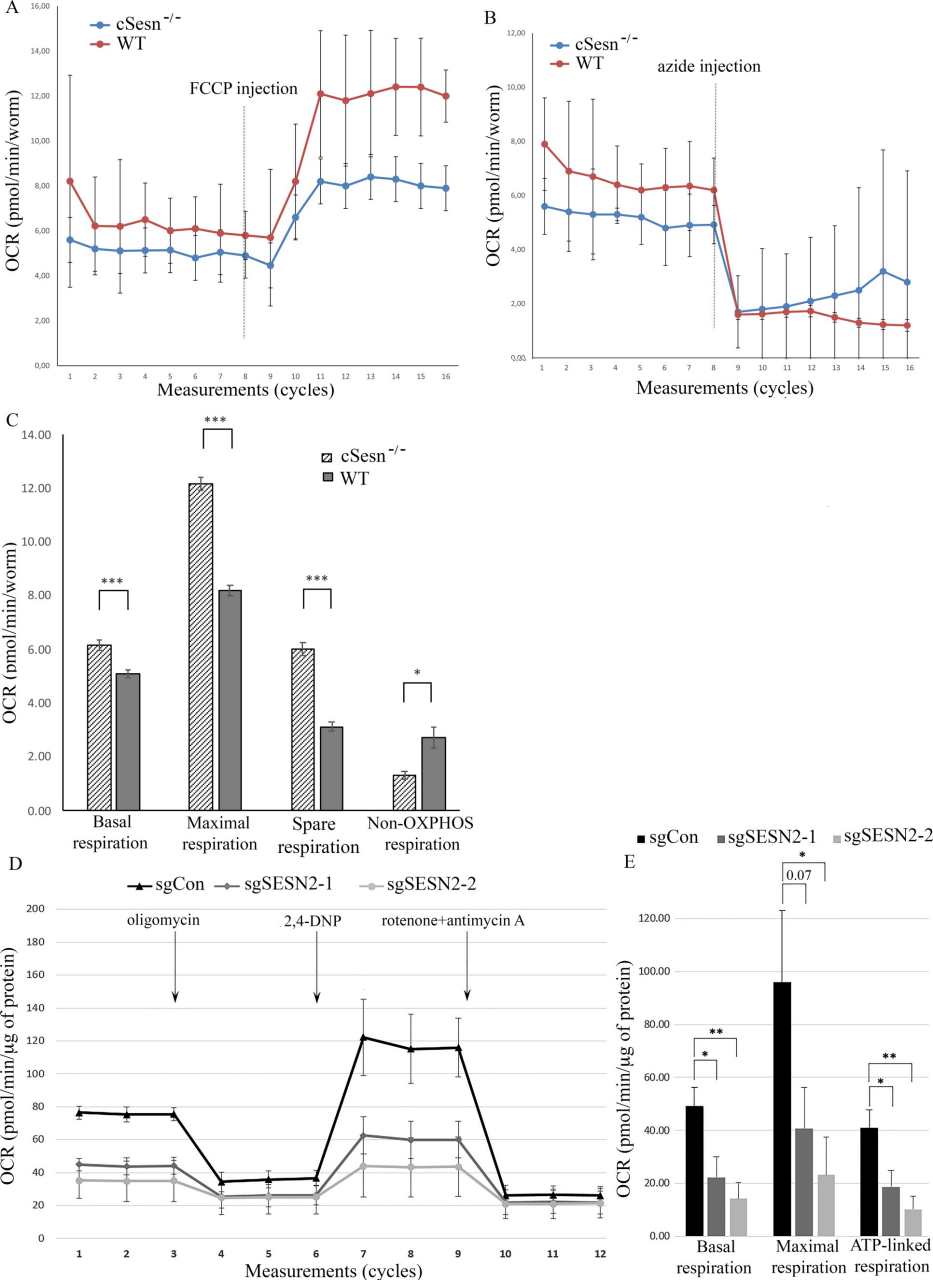
Sestrins support mitochondrial respiration in *C*. *elegans* and adenocarcinoma A549 cells. **(**A) Basal and maximal oxygen consumption rates (OCR) in control (WT) and *cSesn*^*-/-*^ animals. The OCR in the animals was analyzed under normal condition and after injection with FCCP. (B) Basal and residual OCR after treatment with sodium azide. (C) Results of an analysis of basal respiration rate, maximal respiration rate, spare respiration rate, and non-OXPHOS respiration in control and *cSesn*^*-/-*^ animals. The results were normalized according to the number of animals, and the data are presented as means from 5 technical replicates ±SD. Statistical comparisons of the two mean values were estimated using paired two-tailed Student’s t-test, p-value legend is *p≤0.05, **p≤0.01, ***p≤0.001. (D) Basal and maximal OCR in control A549 cells (sgCon), and cells where SESN2 was knocked out by two different CRISPR/Cas9 constructs. (E) Analysis of basal respiration, maximal respiration, and ATP-linked respiration in control and SESN2-null cells. The data were normalized by total protein amount and are presented as means from 3 independent experiments ±SD. Two-tailed Student t-test was used (*p≤0.05, **p≤0.01, ***p≤0.001).

Being associated with mitochondria, SESN2 and other members of the sestrin family might be involved in the regulation of mitochondrial functions such as respiration, ROS production, and cell death [40]. To analyze the potential impact of sestrins on respiration, we analyzed the oxygen consumption rate in the cSesn-proficient and cSesn-deficient *C*. *elegans* strains that were used in our recent studies [5]. We observed that the basal oxygen consumption, maximal respiratory capacity, and spare respiratory capacity observed in the presence of an uncoupler FCCP were considerably higher in WT *C*. *elegans* as compared to cSesn-deficient nematodes. Still, the non-OXPHOS oxygen consumption in cSesn knockout animals was lower than in control animals, as indicated by the treatment with an inhibitor of mitochondrial OXPHOS sodium azide [41] confirming the effects of cSesn on mitochondrial respiration (Fig 6A–6C). To analyze whether SESN2 plays a role in the regulation of mitochondrial respiration in adenocarcinoma A549 cells, we analyzed the levels of basal and maximal respiration in control and SESN2 knockout cells where expression SESN2 was inactivated with two different sgRNA [42]. Inactivation of SESN2 notably decreases the basal respiration rate, the maximal respiration rate, and the respiration rate associated with ATP production in A549 cells indicating the important role of SESN2 in support mitochondrial activity in cancer and normal cells.

## Discussion

Mitochondria play multiple roles in the cells being responsible for life-and-death decisions. Mitochondrial respiration is the primary source of ATP, but this process is also associated with leakage of electrons from the respiratory chain that leads to the production of superoxide and other types of ROS [43]. Although at moderate levels, ROS might play some signaling roles stimulating regulatory pathways involved in cell proliferation [44], exuberant accumulation of ROS causes damages to different types of macromolecules. It compromises different physiological processes that can lead to senescence and cell death [45, 46]. Besides, mitochondria are responsible for the induction of programmed cell death or apoptosis through the accurately orchestrated mechanisms involving the release of mitochondrial proteins, the formation of protein complexes called the apoptosomes, and the activation of cysteine proteases–caspases that execute the apoptotic program [47]. Therefore, timely and spatial control of mitochondrial functions is essential for cells to ensure sufficient energy production, tune up metabolism, prevent ROS accumulation, and control cell death. One of the critical mechanisms of such regulation is mitophagy, which monitors dysfunctional mitochondria and drives them to degradation in the lysosomes [48]. There is permanent communication between mitochondrial and cytoplasmic proteins allowing cells to detect and direct dysfunctional mitochondria to degradation in the autolysosomal pathway. The best-known process of monitoring non-functional mitochondria is driven by the kinase PINK1 that is accumulated on the membranes of damaged mitochondria to recruit the ubiquitin ligase Parkin that in turn ubiquitylates mitochondrial proteins and labels them for further degradation in the lysosomes [48]. However, mitophagy is still observed in different tissues of PINK1-deficient mice, indicating that this process can also be regulated through other, yet still obscure mechanisms [49]. Some other proteins that usually function in different cellular compartments can also be associated with mitochondria and regulate mitochondrial functions. For example, such a function was assigned to the transcription factors STAT3 and p53 that are found associated with mitochondria, where they regulate mitochondrial respiration and cell death, respectively [50, 51].

The stress-responsive SESN2 protein regulates a variety of processes in cells, including metabolism, ROS production, and cell viability [1, 6]. Although it was well-established that SESN2 and other members of the sestrin family suppress mTORC1 and work as leucine sensors, their role in cellular physiology is not limited to the regulation of the mTORC1-dependent processes [52]. We and others have shown that sestrins modulate metabolism, cell death, and autophagy/mitophagy in a mTORC1-independent manner [13, 17, 28, 53]. These data are supported by the observations that we often do not see a difference in the mTORC1 activity between control and SESN1/2-deficient cells, while we still observe the precise impact of sestrins on the regulation of cell death and metabolism [13, 17]. It was previously suggested that sestrins perform most of their functions in the cytosol as ectopically-expressed Flag-tagged SESN2 protein was detected mostly in the cytoplasm [20]. However, in recent work it was demonstrated that SESN2 could also be located on mitochondria by means of interaction with the AKAP1 protein, and SESN2 can suppress the activation of mTORC1 by AKAP1 [36]. AKAP1 is located on the outer mitochondrial membrane, where it works as a scaffold protein that binds and regulates activities of several proteins involved in the regulation of cellular signaling pathways such as those mediated by protein kinase A, tyrosine kinase Src, and protein tyrosine phosphatase D1 [54]. As a result, AKAP1 is capable of controlling some mitochondrial functions including respiration, Ca^2+^ homeostasis, and cell death [54].

To resolve the inconsistency between our results and the observations by Rinaldi et al. [36] we performed several fractionation experiments to determine the localization of SESN2 in cells in control conditions and upon incubation in the glucose-free medium or in the medium where glucose is substituted with galactose. Both conditions affect ATP production causing cellular stress. Although cells survive and propagate in the medium supplemented with galactose, they intensify mitochondrial respiration to meet the demand of ATP supply, while prolonged glucose starvation leads to necrotic cell death mediated by the endoplasmic reticulum stress [13]. Using an approach based on ultracentrifugation [31], we demonstrated that roughly half of the SESN2 protein in a cell could be found in the mitochondrial fraction (Fig 1A). Interestingly, incubation of cells with glucose-free medium and galactose-containing medium increases the amount of protein associated with mitochondria. Our observations were confirmed by other studies where the mitochondrial fraction was isolated by the means of incubation of cellular lysates with beads coupled with the anti-TOMM22 antibody (Fig 1B). As TOMM22 is the protein located exclusively on the outer mitochondrial membrane, this approach allows us to isolate a mitochondrial fraction of high purity [33, 34]. The mitochondrial localization of SESN2 was also confirmed by our studies based on the expression of the SESN2-GFP fusion protein (Fig 2). The discrepancy with our previous data showing that the Flag-SESN2 protein was mostly observed in the cytoplasm can be explained by the application of a recombinant construct that makes an expression of SESN2 extremely high permitting detection of the protein all over the cell and masking the portion of the protein that can be explicitly co-localized with mitochondria. In the current studies, we used a recombinant construct that expresses protein under the control of the weak TP53 promoter that allowed us to keep the expression of SESN2 relatively low. Also, the SESN2 protein is fused with GFP at C-terminus, while in the previous studies, we used SESN2 linked with Flag epitope at the N-terminus that might interfere with mitochondria translocation of SESN2 [20].

In our previous studies, we purified the GATOR2 complex as a significant partner of sestrins [20] and in the present work we determined that the endogenous SESN2 protein immunoprecipitated from mitochondrial fraction was associated with Mios. Therefore, SESN2 might regulate mitochondrial functions via the GATOR2 complex. At the same time, we were not able to detect AKAP1 in the complex with SESN2 or the SESN2 protein in the AKAP1-containing complex (Fig 3A and 3B). These data indicate that it is unlikely that AKAP1 is a bona fide SESN2 partner on mitochondria and the previous data demonstrating the interaction between SESN2 and AKAP1 [36] can be explained by the overexpression of the SESN2 and AKAP1 proteins in HEK293 cells that can lead to non-specific binding between the proteins, especially taking into consideration that they might be located close to the outer mitochondrial membrane (Fig 5)[36]. Also, AKAP1 was not detected in our mass spectrometry analysis of SESN2 protein complexes, while we observed the presence of a considerable amount of all GATOR2 proteins in the complex [20, 55]. However, we cannot rule out the possibility that SESN2 and AKAP1 can regulate each other through some indirect mechanisms, and the relationship between these two proteins need to be better characterized. Furthermore, while it was reported that AKAP1 is capable of stimulating the activity of mTORC1, and SESN2 can counteract its effect, we were not able to observe any notable difference in the mTORC1 activity between control and *SESN1-* and/or *SESN2*-deficient cells incubated under standard cell culture conditions. These data indicate no negative effects of SESN1 and SESN2 on the mTORC1 activity despite the proposed role of AKAP1 in this process [35, 36]. We also demonstrated that while SESN2 does not affect GATOR2 localization on the mitochondria, GATOR2 facilitated mitochondrial localization of SESN2, supporting the idea that AKAP1 is probably dispensable for this process (Figs 3 and 4).

The function of GATOR2 in mitochondria is not well-understood. Interestingly, the partner of GATOR2—the protein complex GATOR1 was found associated with mitochondria where it might interact with a regulator of cell death, mitochondrial protein AIF [27]. Therefore, GATOR1 might support AIF release from mitochondria in response to some stress stimuli that may lead to nuclear translocation of AIF and facilitate the induction of cell death [27]. The SEACAT complex, a homolog of GATOR1 in yeast, is also involved in the regulation of mitophagy in yeast and in the maintenance of mitochondria-vacuole contact sites [56]. These observations demonstrate that GATOR1 might have some mTORC1-independent functions in mitochondria, and GATOR2, being a bona fide GATOR1 interactor, might be involved in the negative regulation of these functions [23, 57]. Therefore, SESN2 might be capable of regulating the GATOR1 functions on mitochondria through the control of GATOR2. However, we cannot rule out the possibility that SESN2 might also regulate mitochondrial activities in a GATOR1/2-independent manner. We have previously shown that Sesn2 regulates mitochondrial respiration and ATP production in control conditions and in the conditions of glucose deprivation in mouse embryonic fibroblasts [13]. In this work, we extended our previous observation using cSesn-negative C. elegans and SESN2-null adenocarcinoma A549 cells as alternative models to demonstrate that inactivation of sestrins decreases mitochondrial respiration rate (Fig 6A–6E). The results obtained with the alternative model systems indicate that sestrins play an evolutionarily conserved role in the regulation of mitochondrial respiration. Although the direct effect of sestrins on the regulation of the components of the mitochondrial respiration chain can be considered, the alternative mechanism may involve mitochondrial quality control mediated by the regulation of mitophagy. Accordingly, the role of SESN2 in support of mitophagy was recently reported [53, 58, 59]. The importance of mitochondrial function of sestrins is also supported by the observations that SESN2 is activated in response to inhibitors of the mitochondrial respiratory chain such as rotenone and piericidin A [20, 60]. Therefore, SESN2 might be required to provide a support for the mitochondrial function in the conditions of mitochondrial stress. Another potential mitochondrial function of sestrins might be the regulation of cell death. Accordingly, we demonstrated that SESN2 regulates cell viability supporting cell death in response to DNA damage and cytokines but suppressing cell death in response to ischemia and oxidative stress [7, 11, 17]. The detailed analysis of the mitochondrial functions of sestrins in the future will allow us to better understand the role of sestrins in the control of cellular homeostasis. As mitochondrial dysfunction underlies many human age-related diseases, the protective effects of sestrins against diabetes, infarct, cancer, neurodegenerative diseases, and muscle atrophy [6, 10, 19] can be explained by their support of mitochondrial function and sestrins might be a desirable target for the development of antiaging drugs aimed to improve mitochondrial activities.

## Supporting information

S1 Data (XLSX)

S2 Data (XLSX)

S1 Raw Images (TIF)
