# Supplementary figures and images for: Mitochondrial localization of SESN2
Source: PLoS One. 2020 Apr 14;15(4):e0226862. doi: 10.1371/journal.pone.0226862 (PMC7156099; doi:10.1371/journal.pone.0226862)

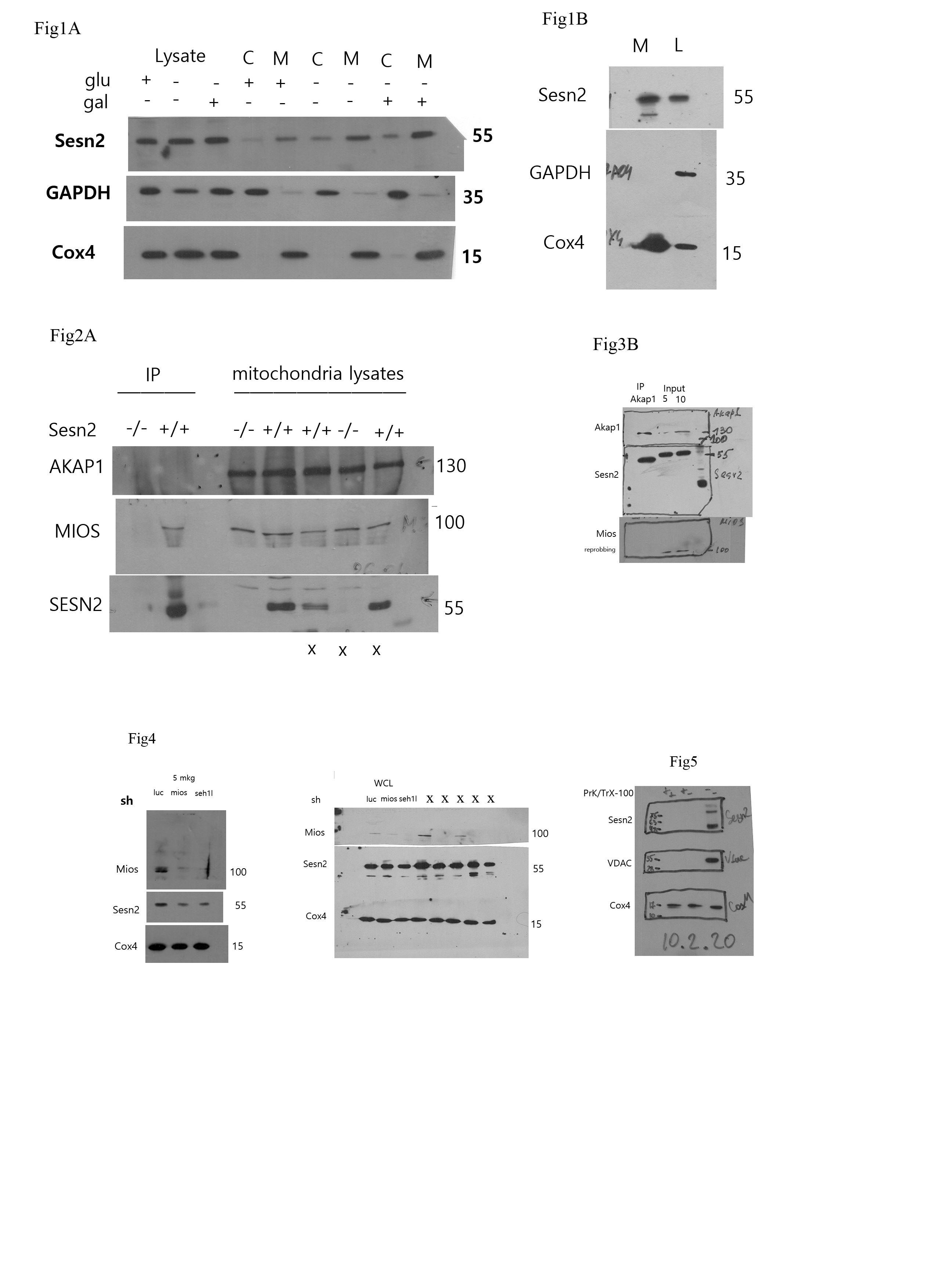

Supplement: S1 Raw Images — (TIF) [file pone.0226862.s003.tif]
